# Immunohistochemically Characterized Intratumoral Heterogeneity Is a Prognostic Marker in Human Glioblastoma

**DOI:** 10.3390/cancers12102964

**Published:** 2020-10-13

**Authors:** Friederike Liesche-Starnecker, Karoline Mayer, Florian Kofler, Sandra Baur, Friederike Schmidt-Graf, Johanna Kempter, Georg Prokop, Nicole Pfarr, Wu Wei, Jens Gempt, Stephanie E. Combs, Claus Zimmer, Bernhard Meyer, Benedikt Wiestler, Jürgen Schlegel

**Affiliations:** 1 Department of Neuropathology, School of Medicine, Institute of Pathology, Technical University Munich, Trogerstraße 18, 81675 München, Germany; karoline.mayer@tum.de (K.M.); sandra.baur@tum.de (S.B.); georg.prokop@tum.de (G.P.); vvwu@stanford.edu (W.W.); schlegel@tum.de (J.S.); 2 Department of Diagnostic and Interventional Neuroradiology, School of Medicine, Technical University Munich, Ismaninger Str. 22, 81675 München, Germany; florian.kofler@tum.de (F.K.); claus.zimmer@tum.de (C.Z.); b.wiestler@tum.de (B.W.); 3 Department of Neurology, School of Medicine, Technical University Munich, Ismaninger Str. 22, 81675 München, Germany; f.schmidt-graf@tum.de (F.S.-G.); Johanna.Kempter@gmx.de (J.K.); 4 Institute of Pathology, School of Medicine, Technical University Munich, Trogerstraße 18, 81675 München, Germany; nicole.pfarr@tum.de; 5 Department of Neurosurgery, School of Medicine, Technical University Munich, Ismaninger Str. 22, 81675 München, Germany; jens.gempt@tum.de (J.G.); bernhard.meyer@tum.de (B.M.); 6 Department of RadiationOncology, School of Medicine, Technical University Munich, Ismaninger Str. 22, 81675 München, Germany; stephanie.combs@tum.de; 7 TranslaTUM (Zentralinstitut für translationale Krebsforschung der Technischen Universität München), Einsteinstraße 25, 81675 München, Germany

**Keywords:** glioblastoma, glioma, molecular pathology, prognostic marker, immunohistochemistry, methylation assay, heterogeneity, relapse, therapy resistance

## Abstract

**Simple Summary:**

Intratumoral heterogeneity is believed to contribute to the immense therapy resistance and recurrence rate of glioblastoma. The aim of this retrospective study was to analyze the heterogeneity of 36 human glioblastoma samples on a morphological level by immunohistochemistry. We confirmed that this method is valid for heterogeneity detection. 115 Areas of Interest were labelled. By cluster analysis, we defined two subtypes (“classical” and “mesenchymal”). The results of epigenomic analyses corroborated the findings. Interestingly, patients with tumors that consisted of both subtypes (“subtype-heterogeneous”) showed a shorter overall survival compared to patients with tumor that were dominated by one subtype (“subtype-dominant”). Furthermore, the analysis of 21 corresponding pairs of primary and recurrent glioblastoma demonstrated that, additionally to an intratumoral heterogeneity, there is also a chronological heterogeneity with dominance of the mesenchymal subtype in recurrent tumors. Our study confirms the prognostic impact of intratumoral heterogeneity in glioblastoma and makes this hallmark assessable by routine diagnostics.

**Abstract:**

Tumor heterogeneity is considered to be a hallmark of glioblastoma (GBM). Only more recently, it has become apparent that GBM is not only heterogeneous between patients (intertumoral heterogeneity) but more importantly, also within individual patients (intratumoral heterogeneity). In this study, we focused on assessing intratumoral heterogeneity. For this purpose, the heterogeneity of 38 treatment-naïve GBM was characterized by immunohistochemistry. Perceptible areas were rated for ALDH1A3, EGFR, GFAP, Iba1, Olig2, p53, and Mib1. By clustering methods, two distinct groups similar to subtypes described in literature were detected. The classical subtype featured a strong EGFR and Olig2 positivity, whereas the mesenchymal subtype displayed a strong ALDH1A3 expression and a high fraction of Iba1-positive microglia. 18 tumors exhibited both subtypes and were classified as “subtype-heterogeneous”, whereas the areas of the other tumors were all assigned to the same cluster and named “subtype-dominant”. Results of epigenomic analyses corroborated these findings. Strikingly, the subtype-heterogeneous tumors showed a clearly shorter overall survival compared to subtype-dominant tumors. Furthermore, 21 corresponding pairs of primary and recurrent GBM were compared, showing a dominance of the mesenchymal subtype in the recurrent tumors. Our study confirms the prognostic impact of intratumoral heterogeneity in GBM, and more importantly, makes this hallmark assessable by routine diagnostics.

## 1. Introduction

Tumor heterogeneity is now considered as a hallmark of glioblastoma (GBM). It is believed to strongly contribute to therapy resistance and accordingly, to the poor prognosis of this tumor entity [1]. To understand the various pheno- and genotypical characteristics and their biology, numerous approaches to subclassify glioblastomas exist. Originally, they were developed to describe intertumoral heterogeneity. Subtypes, called “classical”, “mesenchymal”, “proneural”, and “neural” have been consistently discussed since Verhaak et al. stated that the subtypes show different clinical courses and biology [2]. However, it has become apparent that, even more importantly, heterogeneity within a single tumor exists: Several studies have demonstrated that one tumor can harbor multiple subclones, which are assigned to different subtypes by their molecular characteristics [3,4]. Similar to evolutional processes, diversity leads to advantage. There are different hypotheses regarding the development of heterogeneity in tumors. Based on Charles Darwin’s theory of evolution [5], the hypothesis of clonal evolution sees heterogeneity as a result of natural selection [6]. Genetic instability of tumor cells results in accumulated mutations leading to genetic diversity and heterogeneous morphology. By selective stress, e.g., caused by chemo- or radiotherapy, only adequately adapted cell clones survive [7]. In contrast, the stem cell model acts on the assumption of a hierarchical organization of tumor cells [8]. By self-renewal of stem cell like neoplastic cells, genetically and phenotypically diverse daughter cells develop, from which different intratumoral subtypes arise [9,10]. Besides these two main hypotheses, it is assumed that tumor heterogeneity is a consequence of a multifactorial process, including epigenetic alterations [11], intercellular communication, and interaction with the surrounding microenvironment [10,12]. In addition to regional heterogeneity, a chronological heterogeneity can also be observed when comparing pairs of primary and recurrent GBM. The mesenchymal subtype seems to be most the therapy resistant, since its occurrence increases in recurrent tumors [13]. Most studies on heterogeneity of GBM are based on large-scale genomic characterization. This is a powerful tool for discovery and in-depth tumor analysis, but it has a limited availability. There have been previous attempts to capture heterogeneity in GBM by immunohistochemistry, but these studies focused mainly on intertumoral heterogeneity, though [14,15]. Just recently, we published our first study with a morphological approach by using immunohistochemistry. We defined different tumor regions including region of hypoxia and stem cell region [16]. In this current study, we, again, chose the broadly applicable technique immunohistochemistry, but focused on applying the established subtypes on human tumor tissue with the aim to prove that immunohistochemistry is a valid method for detecting these diverse subtypes in an individual tumor. Furthermore, we hypothesized that the detection of different subtypes within one tumor has impact on its biological and clinical behavior. For this study, markers were chosen that have already been proposed for the recognition of different subtypes. Alterations of the epidermal growth factor receptor (EGFR) are very common in GBM [17,18]. The status of EGFR amplification correlates with the tumor’s potential to migrate [19]. The upregulation of EGFR is a characteristic of the classical subtype, according to Verhaak et al. [2]. The glial fibrillary acidic protein (GFAP), an astrocytic intermediate filament, is associated with migration and motility of astrocytes [18]. The mouse models showed that GFAP-positive tumors act out a more aggressive growth [20]. Oligodendrocyte lineage factor 2 (Olig2) is a transcription factor regulating proliferation of stem cells in the central nervous system (CNS) [21]. In tumors, Olig2 abrogates the proliferation inhibition of tumor suppressor p21 [22]. Experimental Olig2 deletion led to a shift from a proneural to the mesenchymal GBM subtype with the abrogation of EGFR [23]. 25% of primary GBM exhibit alterations in the function of p53 [24]. Mutations of this transcription factor were considered to be characteristic for the proneural subtype of GBM by Verhaak et al. [2]. It has to be noted, though, that the proneural subtype was also defined by mutations of isocitrate dehydrogenase (IDH), which leads to the challenge of whether this assignment is still contemporary. Nevertheless, p53 is of high interest and it was included in this study. In addition, expression of the enzyme aldehyde dehydrogenase 1A3 (ALDH1A3) was analyzed. By its catalytic activity, which leads to oxidation from all-trans retinal to retinoic acid [25], it influences cell proliferation, differentiation, and apoptosis [26]. Furthermore, enzymes of the ALDH family counteract oxidative stress and therefore, protect from cell damage by aldehyde oxidation [27]. As tumor marker, ALDH1A3 is associated with poor outcome in a diversity of malignant tumors, amongst others also in high-grade gliomas [28]. It was shown that the enzyme is associated with the mesenchymal subtype in GBM [26]. Because not only tumor cells constitute tumor tissue, we chose to include ionized calcium binding adaptor molecule 1 (Iba1) as marker of microglial cells, which, on average, reach a fraction of 30 to 40% of all cells in the tumor area [29]. Besides immunosuppressive effects [29], microglial cells promote cell proliferation and migration by the secretion of growth factors [28]. Furthermore, a high amount of microglial cells is associated with the mesenchymal subtype of GBM [13]. Lastly, proliferation marker, molecular immunology borstel 1 (Mib1), was included in this study in order to examine whether proliferation activity was associated with certain GBM subtypes.

By staining the mentioned markers immunohistochemically, this study demonstrates the intratumoral heterogeneity in human glioblastoma samples on a regional level and by also comparing pairs of primary and recurrent GBM on a chronological level.

## 2. Results

### 2.1. Areas of Interest (AoI)

For the primary glioblastomas, a total of 115 AoI were defined, ranging from 1 to 8 AoI per tumor. A total of 80 AoI were determined in the recurrent tumors, also ranging from 1 to 8 per tumor. The sample size of primary tumors varied between 12.5 mm^2^ and 658.0 mm^2^ (mean: 376.0 mm^2^); in the recurrent tumors, the range was from 17.8 mm^2^ to 658.1 mm^2^ (mean: 372.8 mm^2^).

### 2.2. Correlation Analysis

Spearman-rho correlation analysis showed a significant positive correlation between ALDH1A3 expression and GFAP (*r* = 0.228; *p* = 0.014) and Iba1 positivity (*r* = 0.468; *p* = 0.000). GFAP showed strong negative correlations with EGFR (*r* = −0.291; *p* = 0,002), Olig2 (*r* = −0.345; *p* = 0.000), and proliferation marker Mib1 (*r* = −0.413; *p* = 0.000). Furthermore, Mib1 showed a positive correlation with Olig2 (*r* = 0.415; *p* = 0.000). Table 1 shows all of the values. For the actual values of immunohistochemistry, also see Table S1.

### 2.3. Tumor Cells Express ALDH1A3

**Figure 1:**
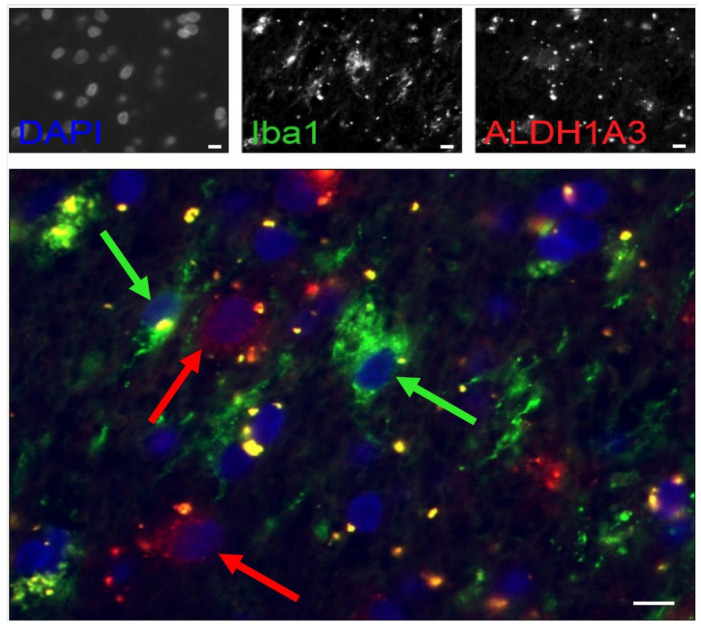
Iba1 and ALDH1A3 immunofluorescence double staining. The picture shows that most cells do not exhibit a co-expression of Iba1 and ALDH1A3. Green arrows mark Iba1-positive microglial cells that lack red signal for ALDH1A3. Red arrows point to ALDH1A3-positive cells without green signal for Iba1. Scale bars: 10 µm.

Correlation analysis showed a strong correlation between Iba1 positivity and ALDH1A3 expression. We performed an immunofluorescence co-staining for Iba1 and ALDH1A3 to exclude that only Iba1-positive microglial cells express ALDH1A3. It can be nicely shown that many ALDH1A3-positive cells do not express Iba1 (Figure 1) and, hence, are not microglial, but assumingly tumor cells.

### 2.4. Cluster Analysis Defines Two Immunohistochemical Subtypes

**Figure 2:**
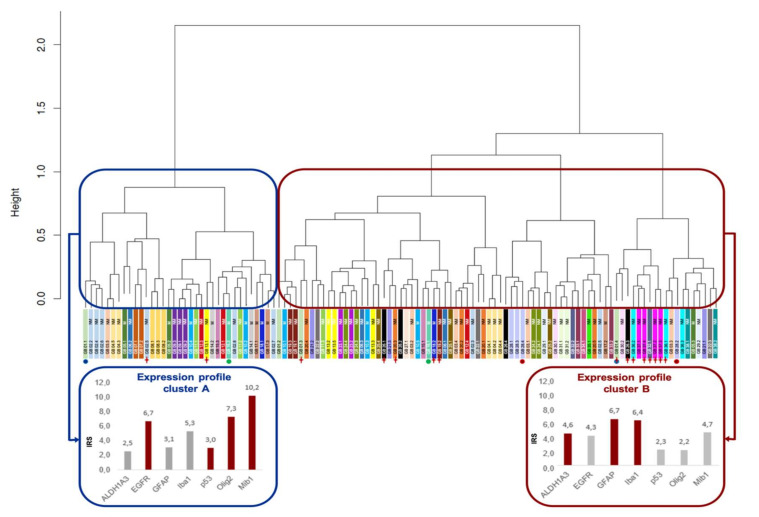
Hierarchical clustering divides the tumor areas into two subtypes. The blue frame is used for cluster **A**, which is comparable to the classical subtype by Verhaak et al. [2], the red frame marks cluster **B** with characteristics of the mesenchymal subtype. In the lower section of the figure, the characteristic immunohistochemical profiles of both clusters are pictured as bar charts. Red bars are used for markers that exceed the average immune reactive score (IRS). The stripes directly below the hierarchical tree with the same color represent the different AoI of the same tumor. In these stripes, information about O-6-methylguanin-DNA methyltransferase (MGMT) promotor status can be found (M = methylated, NM = non-methylated). The dots under the different colored AoIs samples, which consist of pairs of three colors, mark the AoIs that were analyzed epigenetically (the same colors were used in Figure 5b). The red crosses mark the AoIs, whose cluster assignment differs in partitioning around medoids (PAM).

Two clustering methods—hierarchical clustering and partitioning around medoids clustering (PAM)—were used. For both methods, best division was observed for a number of two clusters. Figure 2 shows the hierarchical clustering with the mean scores of all AoI, which were assigned to the two clusters.

**Figure 3:**
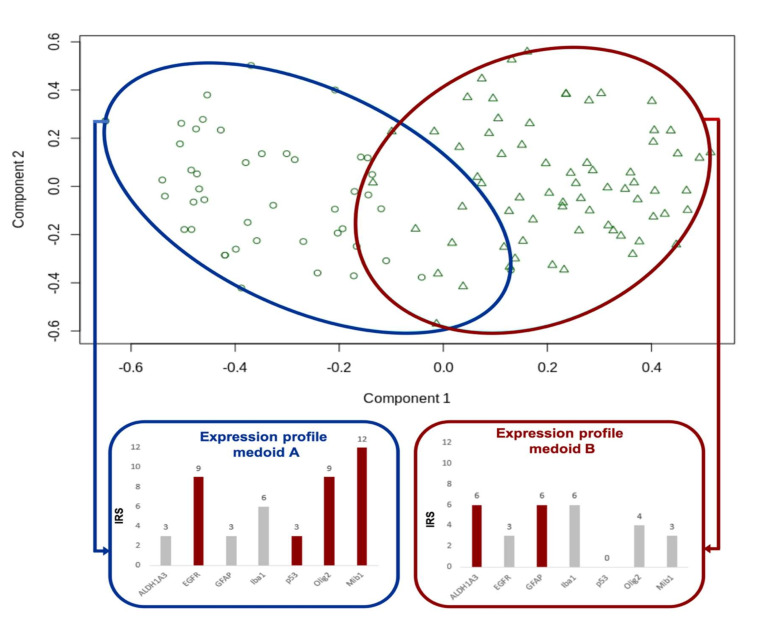
PAM divides the tumor areas into two subtypes. Similar to Figure 2, the blue frame is used for the classical subtype, the red for the mesenchymal subtype. The bar charts show the immunohistochemical expression profiles of the medoids, the samples with the most characteristic immunohistochemical profile of each cluster. Red bars are used for markers that exceed the average (immune reactive score) IRS.

The results were corroborated with PAM analysis (Figure 3). This method also detects a medoid for each cluster, i.e., the sample with the most characteristic immunohistochemical profile of each cluster. These profiles were very similar to those of the mean scores of the two clusters that were achieved by hierarchical clustering. We chose the PAM results for further analysis as (a) calculating the medoids gives an intuitive insight into the “most typical” member of each group and (b) through distance calculation, assigning new samples to one of the two clusters is very straightforward.

The two clustering methods assigned 100 of the 115 (87.0%) defined to the concordant cluster, whereas, for 15 (13.0%) areas, the attribution deviated. Figure 2 marks the divergent AoI.

Through this cluster analysis, two immunohistochemical clusters or subtypes can be described:

#### 2.4.1. Cluster A = Classical/Proliferating Subtype

**Figure 4:**
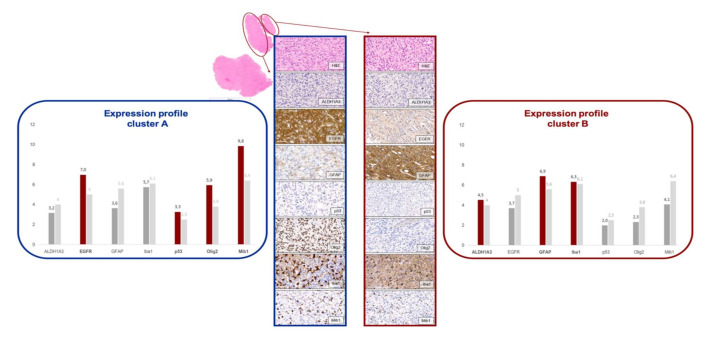
Characteristic immunohistochemical profile of both subtypes. The blue frame marks the classical subtype, the red the mesenchymal subtype. For each, the average IRS of all markers are pictured in the bar charts. Basis is the cluster assignment of PAM. Red bars are used for markers that exceed the average IRS. The light grey bars depict the average IRS for all AoIs.

Forty-six AoI of 23 tumors were assigned to cluster A by PAM clustering. The areas of this cluster show a high proliferation index (mean Mib1 value 38.8%, range 20–90% compared to mean Mib1 value of Cluster B of 15.7%, range 5–90%), which justifies the additional name proliferating subtype in our sample collective. The cluster is further characterized by high expression of EGFR, p53, and Olig2. The positive correlation of these three markers collaborate the profile observation (see also Table 1). EGFR is a well-known marker of the classical subtype of the TCGA classification by Verhaak et al. [2]. In cluster A, the mean immune reactive score (IRS) adds up to 7.0 as compared to an average of 5.0, taken all AoI together. The strong EGFR positivity of the classical/proliferating subtype suggests a close relation to the classical subtype described by Verhaak et al. [2]. Furthermore, Olig2 shows a higher IRS in this cluster (IRS 5.9 compared to an average of 3.8). For the expression profile of the classical/proliferating subtype, see also Figure 4.

#### 2.4.2. Cluster B = Mesenchymal/Microglial-Dominant Subtype

PAM clustering assigned 69 AoI originating from 33 tumors to cluster B, which is defined by the high expression of biomarkers ALDH1A3 (mean IRS of 4.5 as compared to an average of 4.0) and GFAP (mean IRS of 6.9 compared to an average of 5.6). The high amount of Iba1-positive microglial cells (mean IRS of 6.3 as compared to an average of 6.1) led to the additional name microglial-dominant subtype. While in cluster A, proliferation processes seem to prevail, cluster B shows microenvironmental changes. Because ALDH1A3 is a mesenchymal marker, this cluster bears the name mesenchymal subtype, as known from *Verhaak’s* TCGA classification [2]. For the expression profile of the microglial-dominant/mesenchymal subtype, also see Figure 4.

### 2.5. Cluster Analysis Shows Intratumoral Heterogeneity

With cluster analysis, we could confirm the existence of intratumoral heterogeneity, as of the 38 primary GBM, 18 (47.4%) contained AoI, which were assigned to different clusters by PAM. These tumors were named subtype-heterogeneous (ST-het). Of 15 (39.5%) GBM, all intratumoral defined AoI were assigned to the microglial-dominant/mesenchymal subtype. Five (13.2%) tumors consisted of only AoI of the classical/proliferating subtype. Tumors, whose AoI were all assigned to one cluster, were summarized to a subtype-dominant (ST-dom) group.

The mean area of *ST-het* tumors was not significantly larger than the mean area of ST-dom tumors (433.84 mm^2^ compared to 323.85 mm^2^, *p* = 0.096).

### 2.6. Patients with Subtype-Heterogeneous Tumors May Have a Poorer Survival Than Patients with Subtype-Dominant Tumors

**Figure 5:**
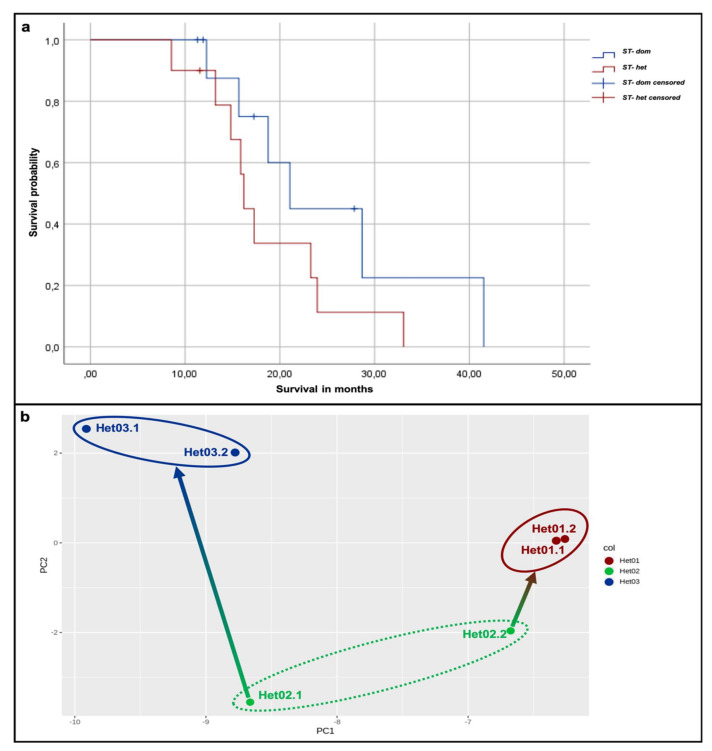
Survival analysis and principal component analysis (PCA) of epigenetic examination. (**a**): By Kaplan–Meier method, a shorter survival of patients with ST-dom tumors compared to patients with *ST-het* tumors can be observed (*p* = 0.166). (**b**): The methylation profile of each two different AoI of three tumors was examined. PCA revealed a proximity of the both samples of tumors Het01 and Het03. Interestingly, the methylation profiling confirms the allocation of the samples of tumor Het02 to the two different clusters by PAM, as one AoI shows a distinct nearer proximity to the samples of Het01 as to the other AoI of the same tumor sample.

The clinical relevance of the two immunohistochemical subtypes and their coexistence in one tumor is shown by the results regarding correlation with clinical data. For survival analysis, only glioblastomas with non-methylated promotor of the O-6-methylguanin-DNA methyltransferase (MGMT) were taken into account to allow for a comparable precondition. The tumor samples tested for their MGMT promotor status are marked in Figure 2. Including only tumors with non-methylated MGMT promotor status led to an analysis of 20 tumors, whereof 10 were assigned to ST-het and 10 to ST-dom. Figure 5a shows the result of the Kaplan–Meier method. Even if it did not reach level of significance (*p* = 0.166), a difference of the two groups can be seen. Patients with ST-het tumors show a shorter overall survival (OS) with a mean OS of 18.6 months (95 % CI 13.9 and 23.2 months) when compared to patients with ST-dom tumors with a mean OS of 25.3 months (95 % CI 16.7 and 33.8).

### 2.7. Epigenetic Profiles Confirm Existence of Intratumoral Heterogeneity

Immunohistochemistry demonstrated intratumoral heterogeneity on protein level. Subsequently, analyses of methylation profiles of tumor samples were conducted in order to confirm this observation. For that, DNA from each two different AoI of three tumors was extracted and examined for their methylation profile using an epigenome-wide EPIC array. For one tumor, the AoI were assigned to the different clusters defined by PAM clustering (sample Het02), whereas, for the other two, both AoI were assigned to the same cluster (Het01 to mesenchymal/microglial-dominant subtype; Het03 to classical/proliferating subtype). The included samples are also marked in Figure 2. There, it can be noted that, by hierarchical clustering, the chosen AoI of Het03 were assigned to different clusters. Despite that, this particular tumor had been chosen for epigenetic profiling due to the appropriate size of AoI for sufficient DNA extraction. Principal component analysis (PCA) of the six samples revealed an expected proximity of the both samples of tumors Het01 and Het03. Interestingly, the methylation profiling confirms the allocation of the samples of tumor Het02 to the two different clusters, as one AoI shows a distinct nearer proximity to the samples of Het01 as to the other AoI of the same tumor sample (Figure 5b).

### 2.8. Dominance of Mesenchymal/Microglial-Dominant Subtype in Case of Recurrence

Intratumoral heterogeneity is a local phenomenon, but it has also a temporal component. This can be demonstrated by comparing primary tumor and its relapse. Twenty-one corresponding pairs of primary and relapse were analyzed and compared regarding their cluster assignment. For the recurrent tumors, a clear dominance of the mesenchymal/microglial-dominant subtype was observed.

**Figure 6:**
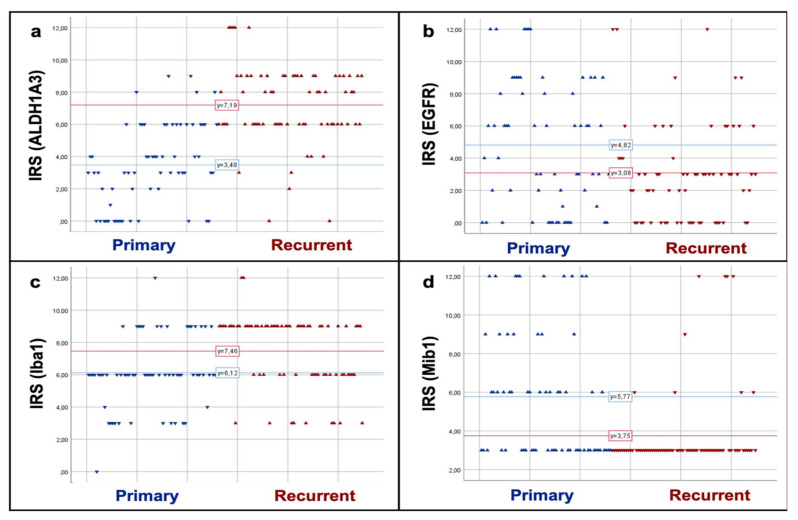
Comparison of marker expression in primary and recurrent GBM. The charts show a strong increase of ALDH1A3 (**a**) and Iba1 (**c**) positivity between primary and recurrent tumors. In contrast, EGFR (**b**) and proliferation (Mib1; **d**) decrease.

Seventy-four (92.5%) of the 80 AoI were assigned to this subtype by PAM clustering, whereas only six (7.5%) were grouped into the classical/proliferating subtype. When only taking in account the recurrent tumors with corresponding primary, 69 of 72 AoI (95.8%) were clustered into the mesenchymal/microglial-dominant subtype, whereas only three (4.2%) were assigned to the classical/proliferating subtype. This led to an assignment of 19 of 21 (90%) recurrent tumors to ST-dom, whereof all were mesenchymal/microglial-dominant. The remaining two (10%) tumors belonged to the *ST-het* group. Figure 6 demonstrates the expression levels of ALDH1A3 (a), EGFR (b), Iba1 (c), and Mib1 (d) in AoI of primary tumors when compared to recurrent tumors. Mean IRS for ALDH1A3 and Iba1, both markers for the mesenchymal/microglial-dominant subtype were much higher in the recurrent tumors compared to the primary tumors (mean IRS for ALDH1A3: 7.19 vs. 3.48; mean IRS for Iba1: 7.46 vs. 6.12). In contrast, EGFR and Mib1, both markers for the classical/proliferating subtype, decreased between primary and recurrent tumors (mean IRS for EGFR: 4.82 vs. 3.08; mean score for Mib1: 5.77 vs. 3.75). T-test analyses showed a significant increase of ALDH1A3 and Iba1 in the progression (*p* = 0.000 and *p* = 0.001) and significant decrease of EGFR and amount of proliferating cells (*p* = 0.005 and *p* = 0.000).

## 3. Discussion

This study confirms the existence of intratumoral heterogeneity in GBM and its influence on the clinical outcome. So far, mostly single cell analyses identified intratumoral heterogeneity on genomic level [4], but, as this study shows, morphological examination, including immunohistochemical protein labeling, also serves as valid heterogeneity detection system. Therefore, our results make assessing this central oncogenetic property of GBM broadly clinically available. Most previous immunohistochemical studies focused on intertumoral heterogeneity. Conroy et al. [14] for instance, defined three subtypes with high expression of EGFR defining the classical subtype. Popova et al. [15] also defined a mesenchymal and a classical subtype. With immunohistochemical analyses of EGFR, PDGFRA, and p53, Le Mercier et al. [30] succeeded with a division in a proneural, characterized by PDGFRA and p53 positivity and a classical subtype with high EGFR expression, as well as “others”, which do not express any of the markers. All of these results are in accordance with our findings, which go beyond that by showing that these subtypes can not only be found in different GBM, but also coexist in one tumor. A third subtype, the proneural subtype, which was proposed by several groups, was mostly defined by IDH mutation [14,15,31] and it should not be compared to IDH wildtype GBM according to the current state of knowledge. In early heterogeneity studies, a neural subtype was named [2]. Newer studies propose the influence of contamination with surrounding normal brain tissue, since this subtype was mostly found at the tumor border [13]. In a recent study, we began to apply the concept of intratumoral heterogeneity by immunohistochemistry and defined different tumor regions, e.g., region of hypoxia, proliferative region and stem cell region [16]. Although, the current study focused on applying the established subtypes on human tumor tissue. By cluster analysis, two subgroups were defined. The first subtype is comparable to the classical subtype by Verhaak et al. [2]. Our results show that it is immunohistochemically characterized by a strong positivity for EGFR and Olig2, as well as a high proliferation activity. High EGFR expression leads to angiogenesis and invasion [9,32]. A heterogenous protein expression has already been described for GBM [33], and it may influence the tumor cells’ potential to migrate [19]. Stem cell marker Olig2, which is also associated with the classical subtype in this study, is integrated in regulating stem cell proliferation [22] and driving tumor growth [34]. Contrary to our results, the transcription factor was shown to be associated with the proneural subtype [30,35], but, as mentioned before, it is questionable if this subtype exists. This might explain why an association between EGFR and Olig2 and their belonging to the same subtype have not been described before. Similar applies for p53 that was also mentioned as characteristic for a proneural subtype before [2,14], and that belongs to the classical subtype in our sample collective. The other subtype, which was defined in this study, is comparable to the mesenchymal subtype by Verhaak et al. [2]. Here, a strong positivity for ALDH1A3 and GFAP was observed. Furthermore, there was an above-average amount of microglial cells in this subtype. An association of ALDH1A3 to this subtype has been described before [26,36]. The enzyme’s influence on cell adhesion and tumor invasion and its capability to reduce oxidative stress could contribute to the poorer outcome of this subtype [26,37]. GFAP is also associated with a more aggressive tumor growth and it was mentioned as a characteristic of the mesenchymal subtype before [35]. The high amount of microglial cells in this subtype has a strong influence on the tumor’s microenvironment. Because their number exceed the amount of tumor cells in some tumors or tumor regions, their effect on therapy resistance must be considered. The observed strong negative correlation of EGFR and GFAP in our sample group is notable. Both of the markers have been mentioned as characteristics for miscellaneous subtypes, EGFR for the classical subtype [2] and GFAP for the mesenchymal subtype [35]. We hypothesize that the strong negative correlation could be due to different “strategical focuses” of the tumor cells with strong expression of EGFR in areas of cell proliferation and the strong expression of GFAP in areas of tumor invasion, as proposed before [38].

After defining a mesenchymal and classical subtype by cluster analysis, we could show that in some cases both subtypes can be found in the same tumor. Epigenetic examination confirmed these observations in one specimen. Additionally, survival analysis suggested that tumors that consist of both subtypes, defined as subtype-heterogeneous tumors, have a poorer outcome when compared to subtype-dominant tumors. This underlines the clinical importance of our study. The regional occurrence also has an impact on biopsy planning for therapeutic decision making, as a biopsy only reflects a small part of the tumor and it may not display the whole tumor’s morphology.

Furthermore, this study dealt with tumor development between primary and recurrent GBM. A chronological heterogeneity was also observed. The mesenchymal subtype dominated in the relapses, which suggests that cells of this subtype may have a higher therapy resistance and, hence, are responsible for relapse occurrence. Previous immunohistochemical studies used the term “mesenchymal transition” for this phenomenon [39,40]. However, as shown in our study, the mesenchymal subtype is also found in areas of the primary GBM, which leads to the preference of using the term “mesenchymal dominance” instead.

Even if our results give a clear picture of intratumoral and temporal heterogeneity in GBM, the results demand confirmatory studies with a larger of samples size, especially to substantiate the clinical impact. Broader examination of the epigenetic heterogeneity on a regional level is needed. The advantage of the use of immunohistochemistry is its broad availability and capacity to reflect the tumor’s morphology. This might clear the way to implementation of regional heterogeneity analyses into standard diagnostics. For this purpose, a standardized evaluation, e.g., by means of a defined heterogeneity index, should be developed.

## 4. Materials and Methods

### 4.1. Material

Tumor samples from 38 treatment-naïve IDH wildtype GBM patients (median age at diagnosis: 60 years; 29 male) plus material of 21 corresponding recurrent tumors were included in this retrospective study. According to Bayerisches Krankenhausgesetz, Artikel 27 it is allowed to use patient data for research given that they are anonymous. In the approval of the ethics committee (Bayerisches Krankenhausgesetz) for our study, it is stated that we do not need the patients’ consent for this retrospective study. Additionally, the material of two recurrent glioblastomas alone (median age at diagnosis: 60 years; two male) were analyzed. For patient data, also see Table 2.

The tissue samples, all formalin-fixed and paraffin-embedded (FFPE), derived from the Department of Neuropathology of the Institute of Pathology, Technical University Munich with a period of surgical resection at the Clinic and Polyclinic of Neurosurgery at Klinikum rechts der Isar from 2011 to 2017. Histopathological diagnosis was performed by neuropathologists and it was re-evaluated for this study according to WHO classification of tumors of the central nervous system, 2016 [41]. The study was performed according to the standards of the Helsinki Declaration of 1975 (as revised in 1983) and approved by the local ethics committee (reference number 164/19 S). Tumor samples of 38 patients were analyzed. Of 26 patients, the MGMT promotor status was known. The overall survival was only calculated for patients with non-methylated MGMT promotor.

### 4.2. Immunohistochemistry

For immunohistochemistry, 2 µm thick slides were cut with a standard microtome and dried at 76 °C for 30 min. EGFR, GFAP, Iba1, Olig2, p53, and Mib1 immunostaining was performed using a fully-automated staining system (Ventana BenchMark ULTRA; Ventana Medical Systems; Tucson, AZ, USA). In brief, the slides were exposed to heat-induced epitope uncovering in pH 8.4 buffer at 95 °C for 32 min. The tissue was incubated with H_2_O_2_ as an inhibitor of endogenous peroxidase in order to prevent unspecific bindings of the primary antibody. Afterwards, the slides were charged with anti-EGFR (monoclonal, mouse, dilution 1:50; Clone E30; DakoCytomation Denmark A/S, Glostrup, Denmark), anti-GFAP (monoclonal, mouse, dilution 1:100; Clone 6F2; DakoCytomation Denmark A/S, Denmark), anti-Iba1 (polyclonal, rabbit, dilution 1:500; Wako Pure Chemical Industries, Japan), anti-Olig2 (monoclonal, mouse, dilution 1:100; Clone 211F1.1; Cell Marque, Rocklin, CALIF, USA), anti-p53 (monoclonal, mouse, dilution 1:200; Clone DO-7; DakoCytomation Denmark A/S, Denmark), or anti-Mib1 (monoclonal, mouse, dilution 1:500; Clone MIB-1; VWR, Radnor, PA, USA) antibodies. For antibody detection, 3,3′-diaminobenzidine- (DAB-) based OptiView DAB IHC Detection Kit (Ventana Medical Systems) was used subsequently.

ALDH1A3 immunohistochemistry was manually performed, starting with epitope uncovering in pH 6.0 citrate buffer at 95 °C for 30 min., followed by H_2_O_2_ incubation. Anti-ALDH1A3 antibody (polyclonal, rabbit, dilution 1:600; Thermo Fisher Scientific, Waltham, MA, USA) was incubated overnight at 4 °C. Biotinylated secondary anti-rabbit IgG antibody (Vector Laboratories, Burlingame, CA, USA) in a dilution of 1:400 and subsequently, ABC-reagents (Vector Laboratories, Burlingame, CA, USA) were incubated for 30 min each., followed by DAB-reagent (Agilent, Santa Clara, CA USA).

For all immunostainings, counterstaining with haematoxylin was conducted and positive controls used as quality assurance.

### 4.3. Staining Evaluation

After slide digitalization with Aperio AT2 scanner (Leica biosystems, Wetzlar, Germany), evaluation of immunohistochemistry was performed by neuropathologists using Aperio ImageScope (version 12.3.0.5056, Leica biosystems, Germany). For every immunohistochemical marker, the areas of interest (AoI) were defined for each slide. For that, perceptible either particularly high or low protein expression was labeled for every marker. Afterwards, marked sectors were matched among all stainings of one tumor sample and overlapping zones were defined as AoI. Subsequently, scores for each marker in every AoI were given. Regarding Iba1, EGFR, GFAP, and Olig2, a variant of the established semiquantitative IRS by Remmele and Stegner was used, which consists of a product of scores of staining intensity (0 = no staining, 1 = weak, 2 = moderate, 3 = strong positivity) and percentage of positive cells (0 = 0%, 1 = 1–4%, 2 = 5–50%, 3 = 51–75%, 4 = 76–100%) [35,42]. For ALDH1A3, the score was slightly modified, since the maximum of stained cells only rarely exceeded 50 % (0 = 0%, 1 = 1–10%, 2 = 11–20%, 3 = 21–50%, 4 = 51–100%). For Mib1, only the percentage of positive cells was counted and translated into a score from 0–12 (0 = 0%, 3 = 1–19%, 6 = 20–29%, 9 = 30–39%, 12 = ≥ 40%). For p53, only strong positive cells were counted and scored while taking into account ranges that were suggested by Takami et al. [43] (0 = 0%, 1 = 1–10%, 2 = 11–89%, 3 = 90–100%).

### 4.4. Immunofluorescence Double Staining

Because the AoI mostly showed similar values for Iba1 and ALDH1A3, double staining with immunofluorescence was conducted in order to evaluate whether tumor cells of Iba1-positive microglial cells are ALDH1A3-positive. The pretreatment was conducted analogous to the above described ALDH1A3 immunohistochemistry. Afterwards, a mixture of the primary antibodies (anti-Iba1: monoclonal, mouse, dilution 1:100; Wako Pure Chemical Industries, Japan; anti-ALDH1A3: polyclonal, rabbit, dilution 1:800; Abcam, UK) was incubated over night at 4 °C followed by the secondary antibody (for Iba1: anti-mouse IgG Alexa Fluor 568, donkey, dilution 1:2000; Thermo Fischer Scientific, USA; for ALDH1A3: anti-mouse IgG Alexa Fluor 488, donkey, dilution 1:2000; Thermo Fischer Scientific, USA) for 45 min. Lastly, counterstaining with DAPI (Roche Diagnostics GmbH, Germany) was conducted, followed by covering with Aqua poly Maunt (Polysciences Inc., Warrington, PA, USA). Positive controls served as quality for evaluation, fluorescence microscope Axio Imager.Z2 (Zeiss, Oberkochen, Germany), and software AxioVision (version 4.8, Zeiss, Germany) were used.

### 4.5. 850k Methylation Array

DNA from marked tumor areas were extracted from FFPE material, followed by measuring the DNA concentration using Quibits dsDNA High sensitivity Assay kit (Invitrogen, Waltham, MA, USA) on a QuBit 4 system. DNA was then applied to Illumina EPIC BeadChip (Illumina, San Diego, CA, USA) for methylation analysis, as previously described [44].

For subsequent analysis of the 850k arrays, the BioConductor package “minfi” [45] was used. After initial quality control, standard processing (SWAN normalization, filtering probes with a detection *p* > 0.05) was applied. A set of glioma-specific CpG sites from Ceccarelli et al. [46] was selected for further analysis in order to visualize spatial proximity of samples. Principal Component Analysis of the filtered methylation data was performed, and samples were plotted by the two largest eigenvectors.

### 4.6. Statistical Analysis

Data were analyzed using R (version 3.3.2, R Foundation for Statistical Computing, Vienna, Austria) and IBM SPSS Statistics (version 22.0 IBM, Armonk, NY, USA). Marker correlation was tested by Spearman–Rho correlation analysis. Welch’s t-test and scatterplots served for comparison of primary and recurrent tumors. The connection of tissue size and number of AoI was analyzed by t-test. The Kaplan–Meier method was used for survival probability analysis. Hierarchical and PAM cluster analyses were conducted for the results of immunohistochemistry. Given the non-continuous nature of this data, Gower’s distance was chosen in order to calculate the underlying dissimilarity matrix (using R function “daisy”). Agglomerative hierarchical clustering with Ward’s method for clustering was performed using R function “agnes”. In parallel, PAM was performed for two groups (k = 2) while using R function “pam”. Statistical significance was defined as *p* < 0.05.

## 5. Conclusions

Our results indicate that the immunohistochemical detection of regional and temporal heterogeneity in GBM is feasible and it potentially provides information important for prognosis and therapy resistance. Consideration should be given to implementing immunohistochemical evaluation of tumor heterogeneity into standard neuropathological diagnostics.

## Figures and Tables

**Table 1:** Spearman-Rho correlation analysis.

		ALDH1A3	GFAP	Iba1	EGFR	p53	Olig2	Mib1
**ALDH1A3**	***r*_s_**		0.228 *	0.468 **	−0.064	0.111	−0.048	−0.121
	***p***		0.014	0.000	0.495	0.240	0.612	0.199
**GFAP**	***r*_s_**	0.228 *		0.085	−0.291 **	−0.115	−0.345 **	−0.413 **
	***p***	0.014		0.369	0.002	0.220	0.000	0.000
**Iba1**	***r*_s_**	0.468 **	0.085		−0.090	0.176	−0.131	0.018
	***p***	0.000	0.369		0.338	0.059	0.162	0.848
**EGFR**	***r*_s_**	−0.064	−0.291 **	−0.090		−0.105	0.326 **	0.282 **
	***p***	0.495	0.002	0.338		0.263	0.000	0.002
**p53**	***r*_s_**	0.111	−0.115	0.176	−0.105		0.026	0.298 **
	***p***	0.240	0.220	0.059	0.263		0.783	0.001
**Olig2**	***r*_s_**	−0.048	−0.345 **	−0.131	0.326 **	0.026		0.415 **
	***p***	0.612	0.000	0.162	0.000	0.783		0.000
**Mib1**	***r*_s_**	−0.121	−0.413 **	0.018	0.282 **	0.298 **	0.415 **	
	***p***	0.199	0.000	0.848	0.002	0.001	0.000	

One star indicates significant level on a basis of *p* < 0.05, two stars on a basis of *p* < 0.01.

**Table 2:** Patient data.

Parameter	Number of Patients	Data	
**Age (in years)**	*n* = 38	mean	59
		median	60
		range	27–84
**Sex**	*n* = 38	male	29
		female	9
**MGMT promotor status**	*n* = 26	methylated	6
		non-methylated	20
**Overall survival (in months)**	*n* = 20	mean (95% CI)	22 (17–26)
		median (95% CI)	19 (13–25)

## Supplementary Materials

The following are available online at https://www.mdpi.com/2072-6694/12/10/2964/s1, Table S1: Results of immunohistochemistry.
