# Supplementary material for: Immunohistochemically Characterized Intratumoral Heterogeneity Is a Prognostic Marker in Human Glioblastoma
Source: Cancers (Basel). 2020 Oct 13;12(10):2964. doi: 10.3390/cancers12102964 (PMC7602025; doi:10.3390/cancers12102964)
Supplement: Supplementary file 1 [file cancers-12-02964-s001.pdf]

Supplementary Material: Immunohistochemically Characterized Intratumoral Heterogeneity Is a Prognostic Marker in Human Glioblastoma

Friederike Liesche-Starnecker, Karoline Mayer, Florian Kofler, Sandra Baur, Friederike Schmidt-Graf, Johanna Kempter, Georg Prokop, Nicole Pfarr, Wu Wei, Jens Gempt, Stephanie E. Combs, Claus Zimmer, Bernhard Meyer, Benedikt Wiestler and Jürgen Schlegel

Table S1. Results of Immunohistochemistry.

ID of AoI	ALDH1A3	EGFR	GFAP	Iba1	p53	Olig2	Mib1
GB 01.1	4	8	0	3	0	6	9
GB 01.2	6	12	2	9	0	4	3
GB 01.3	8	9	0	6	0	6	6
GB 01.4	4	4	0	9	8	0	9
GB 02.1	0	0	2	3	0	0	3
GB 02.2	1	6	1	3	0	4	6
GB 02.3	2	6	4	3	0	4	3
GB 02.4	0	6	0	3	0	6	9
GB 02.5	0	2	0	6	0	4	6
GB 02.6	0	9	0	3	0	6	6
GB 02.7	0	9	2	6	0	6	9
GB 02.8	0	9	2	9	3	6	12
GB 03.1	3	0	12	6	3	0	3
GB 03.2	4	4	9	6	3	4	3
GB 03.3	4	0	12	6	3	0	9
GB 03.4	3	6	6	6	0	4	3
GB 03.5	0	12	3	0	3	9	12
GB 04.1	3	3	9	0	0	6	3
GB 04.2	3	3	9	0	0	6	3
GB 04.3	2	12	0	0	3	4	9
GB 04.4	6	9	6	0	0	6	3
GB 04.5	0	12	3	0	3	9	12
GB 05.1	6	0	0	6	8	9	12
GB 05.2	6	4	4	6	3	6	9
GB 05.3	6	4	6	6	3	0	6
GB 06.1	6	2	6	9	8	6	6
GB 06.2	9	1	9	6	8	4	3
GB 06.3	3	0	0	3	12	0	12
GB 07.1	0	0	3	6	3	6	12
GB 07.2	0	0	1	6	0	9	12
GB 08.1	0	4	2	3	8	6	12
GB 08.2	0	4	6	3	8	6	6
GB 08.3	0	4	2	3	8	9	9
GB 09.1	6	6	4	9	3	9	12
GB 09.2	6	6	4	9	3	9	12
GB 09.3	6	4	3	6	3	9	12
GB 10.1	6	6	6	9	0	3	3
GB 10.2	0	9	6	9	3	9	12
GB 10.3	9	2	6	9	3	3	3
GB 10.4	6	6	6	6	3	9	12
GB 10.5	4	6	6	9	3	6	3
GB 11.1	8	12	4	6	3	9	12

GB 11.2	3	12	2	9	3	6	9
GB 11.3	8	6	2	9	3	0	3
GB 12.1	3	9	3	6	3	9	12
GB 12.2	6	9	4	6	3	9	12
GB 12.3	3	8	4	6	0	3	3
GB 13.1	6	3	9	9	0	6	9
GB 13.2	9	0	9	9	0	6	3
GB 13.4	9	0	3	9	0	6	3
GB 13.5	12	3	9	9	0	6	3
GB 14.1	3	8	6	6	8	6	12
GB 14.2	6	6	9	6	3	6	9
GB 15.1	3	2	6	6	3	4	6
GB 15.2	0	6	3	6	3	9	6
GB 16.1	4	3	6	6	0	4	6
GB 16.2	4	9	3	6	3	9	9
GB 17.1	2	12	4	6	0	12	12
GB 17.2	0	4	6	4	0	0	6
GB 17.3	0	8	2	6	0	12	9
GB 18.1	2	8	8	6	0	6	12
GB 18.2	4	6	6	6	3	6	6
GB 19.1	8	9	6	6	0	4	3
GB 19.2	4	3	4	6	3	6	6
GB 19.3	6	9	6	9	0	3	3
GB 20.1	0	12	9	3	0	3	3
GB 20.2	2	12	6	6	0	4	3
GB 20.3	3	12	6	6	3	4	6
GB 20.4	8	12	6	9	0	6	6
GB 20.5	0	0	9	9	0	0	3
GB 21.1	6	4	6	6	0	0	5
GB 21.2	12	8	8	9	3	0	3
GB 21.3	3	9	6	6	3	3	3
GB 22.1	0	8	4	0	0	0	3
GB 22.2	6	9	4	6	3	0	3
GB 22.3	6	0	12	6	0	0	6
GB 23.1	6	1	6	6	0	3	3
GB 23.2	6	3	9	9	0	3	3
GB 24.1	3	0	9	9	3	2	3
GB 24.2	3	0	12	6	3	4	3
GB 24.3	8	3	9	6	0	4	3
GB 24.4	6	3	6	6	0	4	3
GB 25.1	6	0	6	6	3	6	6
GB 25.2	4	0	9	3	3	0	3
GB 25.3	6	0	3	6	3	6	3
GB 26.1	4	9	12	3	3	6	6
GB 26.2	9	6	9	6	3	3	3
GB 26.3	9	9	3	6	12	0	9
GB 26.4	3	3	9	6	8	0	3
GB 27.1	6	6	9	9	3	4	3
GB 28.1	4	0	4	12	8	0	3
GB 28.2	4	0	4	9	3	0	6
GB 28.3	2	0	3	6	8	0	3
GB 28.4	3	3	3	9	12	0	3
GB 29.1	4	0	9	3	0	0	3
GB 29.2	6	0	6	6	3	0	6
GB 30.1	2	2	9	6	0	0	3
GB 30.2	6	3	6	9	12	0	12
GB 31.1	3	3	9	3	0	0	3
GB 31.2	3	3	8	3	0	0	3

GB 32.1	2	9	6	6	3	0	12
GB 32.2	0	3	9	3	0	0	3
GB 33.1	0	6	9	4	0	0	3
GB 33.2	0	9	6	6	0	0	6
GB 34.1	0	0	6	6	3	3	3
GB 35.1	0	0	6	6	0	0	3
GB 36.1	3	0	9	9	3	0	9
GB 36.2	6	6	9	9	3	0	12
GB 36.3	6	3	9	9	3	0	6
GB 37.1	6	3	6	9	3	0	9
GB 37.2	6	8	3	9	0	0	12
GB 37.3	3	8	3	9	0	0	9
GB 37.4	6	9	6	9	3	0	9
GB 38.1	6	3	9	9	3	0	3
GB 38.2	6	2	12	6	0	0	9
**Mean**	**4,0**	**5,0**	**5,6**	**6,1**	**2,5**	**3,8**	**6,4**

Shown are the values of the IRS.
